# Supplementary material for: Effects of Pine Pollen Polysaccharides and Sulfated Polysaccharides on Ulcerative Colitis and Gut Flora in Mice
Source: Polymers (Basel). 2023 Mar 13;15(6):1414. doi: 10.3390/polym15061414 (PMC10058757; doi:10.3390/polym15061414)
Supplement: Supplementary file 1 [file polymers-15-01414-s001.zip › polymers-2176601-supplementary.pdf]

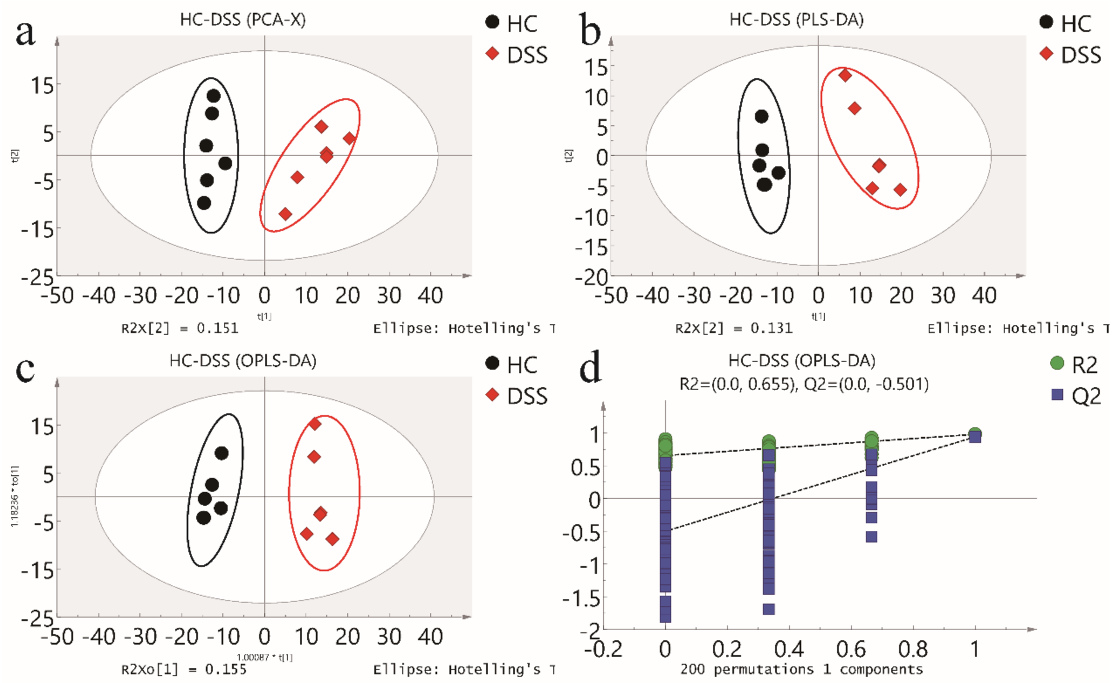

Figure S1. Multivariate statistical analyses of ^{1}H NMR spectra of the HC and DSS groups.

(a) PCA score map; (b) PLS score map; (c) OPLS score map; (d) The validation model of OPLS-DA.

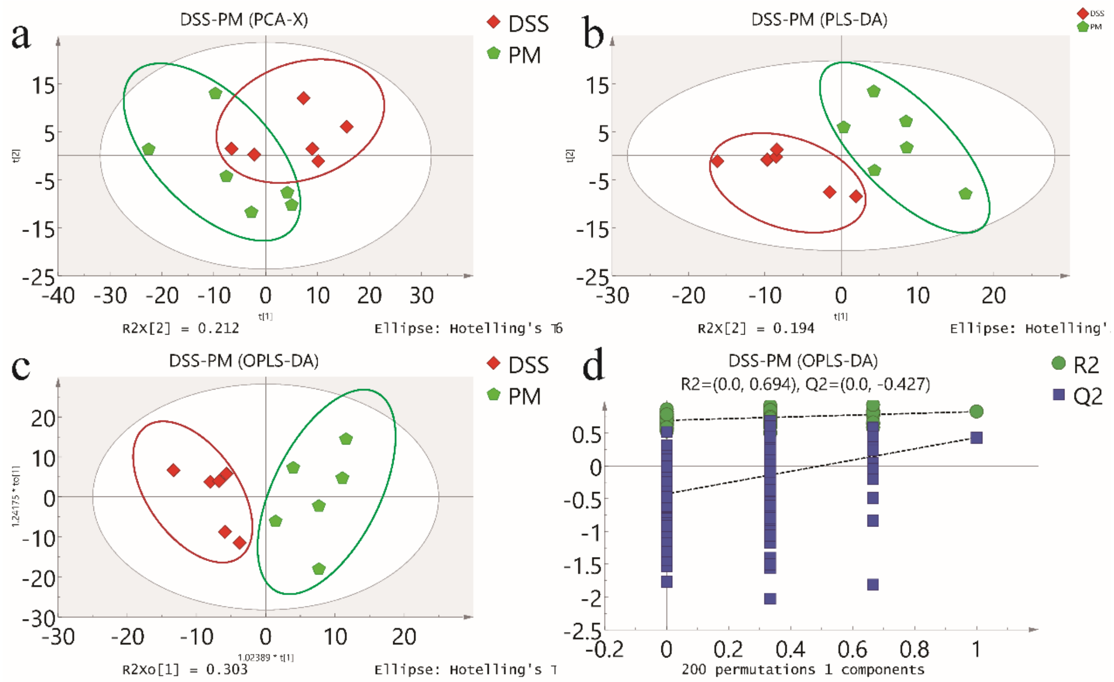

Figure S2. Multivariate statistical analyses of ^{1}H NMR spectra of the DSS and PM groups.

(a) PCA score map; (b) PLS score map; (c) OPLS score map; (d) The validation model of OPLS-DA.

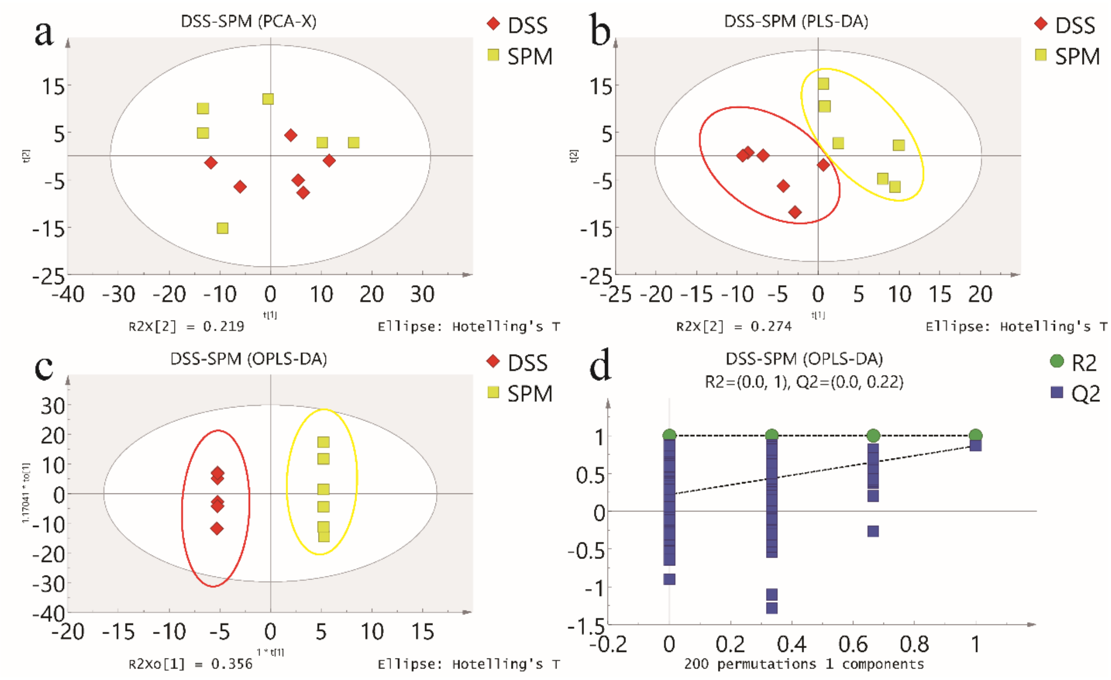

Figure S3. Multivariate statistical analyses of ^1H NMR spectra of the DSS and SPM groups.

(a) PCA score map; (b) PLS score map; (c) OPLS score map; (d) The validation model of OPLS-DA.
